# Highway proximity associated with cardiovascular disease risk: the influence of individual-level confounders and exposure misclassification

**DOI:** 10.1186/1476-069X-12-84

**Published:** 2013-10-03

**Authors:** Doug Brugge, Kevin Lane, Luz T Padró-Martínez, Andrea Stewart, Kyle Hoesterey, David Weiss, Ding Ding Wang, Jonathan I Levy, Allison P Patton, Wig Zamore, Mkaya Mwamburi

**Affiliations:** 1 Tufts University School of Medicine, Boston, MA, USA; 2 Boston University School of Public Health, Boston, MA, USA; 3 Tufts University School of Engineering, Medford, MA, USA; 4 Tufts University School of Arts and Sciences, Medford, MA, USA; 5 Somerville Transportation Equity Partnership, Somerville, MA, USA

**Keywords:** Highway proximity, Air pollution, Traffic, Geocoding, Inflammation

## Abstract

**Background:**

Elevated cardiovascular disease risk has been reported with proximity to highways or busy roadways, but proximity measures can be challenging to interpret given potential confounders and exposure error.

**Methods:**

We conducted a cross sectional analysis of plasma levels of C-Reactive Protein (hsCRP), Interleukin-6 (IL-6), Tumor Necrosis Factor alpha receptor II (TNF-RII) and fibrinogen with distance of residence to a highway in and around Boston, Massachusetts. Distance was assigned using ortho-photo corrected parcel matching, as well as less precise approaches such as simple parcel matching and geocoding addresses to street networks. We used a combined random and convenience sample of 260 adults >40 years old. We screened a large number of individual-level variables including some infrequently collected for assessment of highway proximity, and included a subset in our final regression models. We monitored ultrafine particle (UFP) levels in the study areas to help interpret proximity measures.

**Results:**

Using the orthophoto corrected geocoding, in a fully adjusted model, hsCRP and IL-6 differed by distance category relative to urban background: 43% (-16%,141%) and 49% (6%,110%) increase for 0-50 m; 7% (-39%,45%) and 41% (6%,86%) for 50-150 m; 54% (-2%,142%) and 18% (-11%,57%) for 150-250 m, and 49% (-4%, 131%) and 42% (6%, 89%) for 250-450 m. There was little evidence for association for TNF-RII or fibrinogen. Ortho-photo corrected geocoding resulted in stronger associations than traditional methods which introduced differential misclassification. Restricted analysis found the effect of proximity on biomarkers was mostly downwind from the highway or upwind where there was considerable local street traffic, consistent with patterns of monitored UFP levels.

**Conclusion:**

We found associations between highway proximity and both hsCRP and IL-6, with non-monotonic patterns explained partly by individual-level factors and differences between proximity and UFP concentrations. Our analyses emphasize the importance of controlling for the risk of differential exposure misclassification from geocoding error.

## Background

Residential proximity to major roadways and highways has been found to be associated with numerous adverse health outcomes, including cardiovascular diseases [1-3]. These studies suggest that prior conditions, diabetes and obesity for example, make individuals more vulnerable to traffic exposure [4,5]. Only a few studies have reported levels of blood markers–C-Reactive Protein (hsCRP), Interleukin-6 (IL-6), and fibrinogen–relative to distance to highways or roadways [5-7].

A primary hypothesis for near roadway health effects has been traffic-related air pollutants, many of which are elevated next to high traffic roadways [8]. A recent meta-analysis of near highway air monitoring studies found that there was consistent evidence for steep gradients of UFP, elemental carbon, volatile organic compounds, CO, NO and NO_x_[9]. These pollutants tend to decline to urban background levels within 200-400 m, vary considerably with changes in meteorology, and have most often been measured over short time periods, typically individual days [10]. While health studies have reported exposure to various pollutants as well as distance to roadways [2,7], none have yet assigned exposure to UFP in the near highway environment. With or without pollutant exposure measures, proximity could represent traffic noise, a factor we could not address in this analysis [11], or gradients of socioeconomic status (SES) near heavy traffic, raising the need to carefully address potential confounders.

Prior traffic proximity studies have often used exposure metrics with potentially significant misclassification. Many studies that use proximity as an exposure proxy have assigned residential locations by geocoding addresses to street networks, which introduces positional error that could bias results of fine-scale proximity analysis [12-14]. Previous analysis of this study population found a mean positional error of 39 m and 49 m when geocoding to a commercially and publicly available street network address dataset, respectively [15]. Given steep pollution gradients within 200 m of a highway, this degree of error could be significant.

The Community Assessment of Freeway Exposure and Health study (CAFEH) is a community-based participatory research cross sectional study of near highway air pollutants, primarily UFP, and blood markers of cardiovascular risk [16]. Here we report an analysis of proximity to a major highway and association with blood markers of cardiovascular risk. We focus on state of the art geopositioning of residential addresses and consideration of a large number of potential confounders. We also use UFP concentration patterns to inform stratified analyses that better reflect spatial distributions of pollutants.

## Methods

### Recruitment

**Figure 1:**
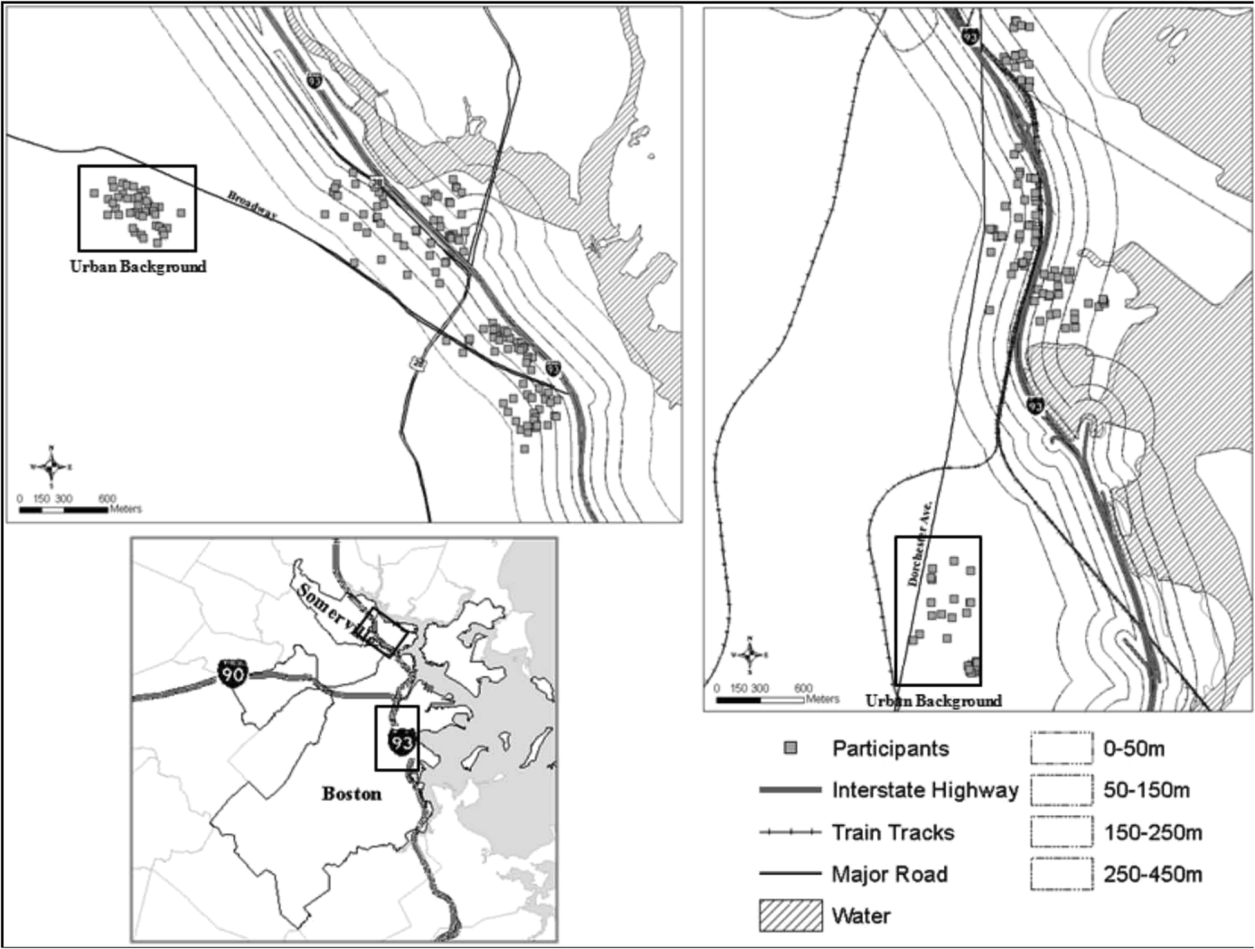
The near highway and urban background study areas in (a) Somerville and (b) Dorchester with participant residential addresses.

The analysis presented here includes data from two near-highway areas and two paired urban background areas, located in Somerville and in the Dorchester and South Boston neighborhoods of Boston, MA [Somerville and Dorchester hereafter; Figure 1[16]. A third neighborhood from which we recruited, Chinatown in downtown Boston, was excluded because the highway geometries and street canyons complicated assignment of simple proximity values. Recruitment proceeded in approximately one year blocks. In each neighborhood we stratified recruitment for <100 m, 100-400 m and >1000 m from the edge of Interstate-93 (I-93) in order to maximize local exposure contrast. We ended up with a small number of residences outside of 400 m so we extended the study to 450 m. On the basis of location of our recruited sample, we excluded from analysis the 450-1000 m areas. All participants in the study areas resided in buildings that were no more than 6 stories high and most were in buildings of 3 stories or less. Random samples were generated for all addresses within our study areas and every address in the random sample was approached. We had complete sets of documents available in English, Spanish, Portuguese, Haitian Creole, Vietnamese and Chinese and field members fluent in these languages to ensure broad inclusion of non-English speaking residents. Recruitment was door-to-door by surveyors who received extensive training and supervision. To bolster numbers, we recruited additional convenience samples. The convenience samples largely consisted of residents in 4 elderly housing developments, 2 each in Somerville and Dorchester. The study protocol and consent forms were approved by the Tufts Health Sciences IRB.

### Human data

Participants who enrolled in the study completed a survey in their home which included questions about demographic information (Table 1). Time activity was collected for 2 recent days and included time spent inside and outside at home, at work/school, at other locations, and on highways for each hour for a recent workday/weekday and non-workday/weekend. Time activity data displayed significant differences in micro-environment time allocation when stratified by demographic variables, but low within participant variability between the first and second questionnaire [15]. We asked questions that assessed exposure to highway pollutants in other microenvironments (residential, occupational, commuting, etc.). We also gathered information on possible confounders with cardiovascular disease (diet, physical activity, stress, etc.) and inquired about relevant diagnosed comorbidities (diabetes, hypertension, etc.). Medications were recorded from labels of all prescriptions that were available in the home and were classified into broad categories by a physician: statins, oral hypoglycemic agents (OHAs), insulin, anti-hypertensives, antacids, anti-inflammatories and hormones. Data were double entered into MS Access, checked for errors and corrected (verified and validated) by reference to the original survey hard copy. Most variables included in the regression models had 1% or less missing. BMI and smoking status had 8% and 4% missing, respectively. Income had the largest percent missing at 11%. Those with missing income were categorized into a separate group and retained in the analysis.

**Table 1:** Characteristics of the study population stratified by categories of distance to the highway

	***Within 50 m***	***50 m-150 m***	***150-250 m***	***250-450 m***	***>1000 m***
	***(N = 30)***	***(N = 58)***	***(N = 65)***	***(N = 54)***	***(N = 53)***
*Demographic variables*					
Age, mean (SD)	56.1 (11)	55.9 (11.36)#	61.9 (10.6)*#	58.6 (10.97)	56.7 (13.65)
BMI	31.9 (7.42)*#	27.7 (5.75)#	31.9 (7.85)*#	29.5 (7.65)	28.2 (7.58)
Female	57%	52%	58%	63%	68%
Born in USA					
Yes	53%	71%	72%	57%	68%
Missing	10%	1%	3%	2%	4%
Race					
White	67%	70%	65%	63%	64%
Non-white	30%	28%	34%	37%	36%
Missing	3%	2%	1%	0%	0%
Annual household income					
Less than $24,999	27%#	26%#	61%*#	35%#	28%
$25,000-$74,999	43%#	40%#	25%#	31%	25%
$75,000 or more	10%	22%#	8%*#	22%#	38%
Don’t know/ refused	20%#	12%	6%#	11%	9%
Terminal degree					
Less than high school	27%	14%	31%	24%	13%
High school	46%*#	33%	26%#	37%	21%
Undergraduate	27%	39%*#	25%#	20%	32%
Graduate	0%	14%	18%	19%	34%
Employment					
Working full or part time	53%#	45%#	28%*#	43%	53%
Retired, disabled, unemployed	37%#	52%#	69%*#	57%	45%
Missing	10%	3%	3%	0%	2%
Study area					
Somerville	67%#	38%*#	53%	69%*#	49%
*Exposure variables*					
Workday time spent inside home (hrs)	14.6 (5.41)	17.1 (4.62)	17.4 (4.16)	16.7 (5.85)	16.8 (4.39)
Non-workday time spent inside home (hrs)	17.2 (5.37)	18.5 (4.23)	19.5 (4.12)	18.7 (5.27)	17.7 (5.20)
Previous week combustion exposure score	3.1 (2.35)	3.9 (1.92)	3.8 (2.03)	3.9 (2.38)	3.2 (1.98)
Job combustion score	3.27 (1.62)	3.21 (1.64)	3.70 (1.65)	3.20 (1.69)	3.17 (1.61)
Open windows in winter					
Yes	59%	59%	63%	52%	49%
Missing	3%	3%	2%	0%	0%
Open windows in summer					
Yes	96%	96%	91%	91%	94%
Missing	3%	2%	5%	0	2%
Travel on highway					
Yes	17%#	4%*#	15%#	17%#	11%
Missing	3%	2%	0%	0%	0%
*Health & medications*					
Statin medication	13%	33%	21%	33%	23%
Previous heart attack	3%	5%	9%	5%	6%
Diabetes	13%	14%	15%	15%	15%

*Indicates a significant mean or proportional difference from the urban background (>1000 m).

#Indicates a significant mean or proportional difference from any other group.

We derived variables for race as white or non-white, based on the small numbers in other racial minority categories. An occupational combustion exposure was based on a qualitative assessment of each participant’s current and past occupation(s) along with self-reported exposure on the job. Pack years of smoking was calculated for current and past smokers. Vigorous leisure time physical activity was calculated based on frequency and duration. Upon completing the in-home survey, participants were invited to attend field clinics (typically within weeks of the home visit) after fasting through the night. Clinics were held in the morning in the study areas. At the clinics, we administered a second brief survey that included illnesses in the past week, alcohol consumption, when they last ate, whether they had recent stressful life events (open ended) and exposure to 18 sources of combustion in the previous week. A combustion exposure score was derived by adding up the number of reported combustion exposures in the week preceding their blood draw.

Height and weight were recorded using a standard scale (SECA, Model #8761321009) and stadiometer (Shorr Productions LLC, Model #905055). Diastolic and systolic blood pressure were measured in the right, then left, then right arms with the participant seated using an automatic blood pressure machine (Model #HEM711ACN2, Omron Healthcare, Kyoto, Japan). Hypertension was defined as either measured elevated blood pressure or taking antihypertensive medications. Blood lipid profile was measured on site from a finger stick using a CardioChek PA device (Polymer Technology Systems, Inc. CardioChek, Indianapolis, IN). A venous blood sample was taken, processed to plasma and stored at minus 80 degrees centigrade. Stored plasma was analyzed in 3 batches. Each sample was assayed using immunoassay kits for hsCRP (SPQ High Sensitivity CRP Reagent Set; DiaSorin, Stillwater, MN); fibrinogen (κ-Assay, Kamiya Biomedical, Seattle, WA); Tumor Necrosis Factor alpha receptor II (TNF-RII; Quantitative, R & D Systems, Minneapolis, MN); and IL-6 (Quantitative HS, R & D Systems, Minneapolis, MN).

Participants with hsCRP levels greater than 10 mg/L (N = 23) were examined for individual/group mean differences for BMI, current smoking, recent illness, serious chronic illness, and recent combustion exposure. We found no trends in individuals or significant group differences in means that could justify removing them from the analysis.

### Geographic data

Residential address and apartment numbers were verified during recruitment. Parcel address geo-databases were obtained from the Somerville and Boston GIS and city planning departments and used to geocode residential addresses of study participants within ESRI ArcGIS 10.1. Aerial photography with 15-30 cm resolution and horizontal error less than 1 m from a 2008–2009 flyover of Massachusetts was downloaded from the Massachusetts Office of Geographic Information and used to manually locate each residence from the parcel centroid to the center of residential buildings (N = 235) [17]. Parcel building and floor plans were obtained for parcels with multiple or larger buildings. Floor plans were scanned and georeferenced to the aerial photos in ArcGIS to assign the apartment within each building. Parcel geocoding with aerial photography has been considered a gold standard methodology for address assignment [14]. To the best of our knowledge this study is the first near highway health study to employ this level of precision.

We defined highways to include entrance and exit ramps as well as feeder roads running parallel to the highway. The state road network contains a surface width variable that was used to create an edge of roadway buffer, which was visually verified for accuracy using the aerial photography layer. Distance to highway was calculated for each residence within ArcGIS by conducting a spatial join to the edge of highway polygon, providing a Euclidian distance. These values were then used to categorize study participants into categories of 0–50 m, 50-150 m, 150-250 m, 250-450 m, and ≥ 1000 m (urban background) from the highway. Distance to highway was explored as a continuous variable, but was found to not be appropriate since there is a gap between 450 m and 1000 m where participants were intentionally not recruited as part of the CAFEH study in order to maximize exposure contrast in the study population. Proximity cut points were determined based upon previous literature identifying strongest association with 0-50 m from a major roadway and cardiovascular health outcomes [6,7]. Subsequent exposure groupings were determined based upon maximizing number of cut points while maintaining sufficient sample size to conduct a multiple linear regression. A dichotomous exposed/unexposed cut point (categories of 0-450 m and 1000 m+) was found to not be significantly associated with hsCRP and IL-6. Other cut points that mixed the 0-50 m category (categories of 0-100 m, 100-450 m and 1000 m+; and categories of 0-50 m, 51-450 m and 1000 m+) also did not show significant associations.

### Air pollution data

Mobile monitoring of particle number concentration which is dominated by UFP was conducted with the Tufts Mobile Air Pollution Laboratory (TAPL), a converted recreational vehicle equipped with a condensation particle counter (TSI Model 3775). The TAPL was driven on the same route which encompassed the areas with study participants for 283 hours in Somerville and 141 hours in Dorchester/South Boston [15,18]. Particle number concentrations, are presented for the distance categories given above. The instrument time stamp was used to correct for measurement lag times (3 seconds). Other details of quality control are reported elsewhere [18]. All the data collected in each distance category listed above is presented. We excluded data collected between 450 m and 1000 m because there were no study participant residences in this range of distances from the edge of I-93.

### Statistical methods

Analyses were performed using SAS^®^ (Statistical Analysis Software, Cary, North Carolina) version 9.12 and SPSS^®^ (SPSS, Inc., Chicago, IL) version 20.0. Bivariate analyses were conducted using t-tests and Wilcoxon tests to compare means and medians for normally and non-normally distributed continuous variables respectively between two categories. Analysis of variance (ANOVA) with a post-hoc Tukey multiple comparisons test were used to compare means of normally distributed continuous variables between the exposed and urban background groups. Differences in medians for non-normally distributed continuous variables for each exposed group and urban background were calculated using Wilcoxon tests with a post-hoc Bonferroni correction for multiple comparisons. Chi-square analysis and Fisher’s exact test, when appropriate, were used to compare differences in proportions. All hypothesis tests were two-sided.

Multivariate regression consisted of examining the association between proximity to highway and lognormal-transformed levels of hsCRP, IL-6 and TNF-RII. The lognormal-transformed regression ß-estimates and 95%CIs were exponentiated to obtain the percent difference between each exposed group and urban background for each outcome. Fibrinogen was normally distributed and was examined for absolute differences.

Model-building involved consideration of variables, using a series of bivariate analyses to identify potential confounders. Age, sex, and smoking status were forced into the models. Variables associated with both the outcome and main predictor which had p-values less than 0.15 were considered potential confounders and included in the multivariate linear regression model building process. Adjusted linear regression model building was performed using a forward stepwise selection approach with a p-value of 0.15 as both entry and exit criteria. We performed an additional manual selection process where variables were retained if they had an impact on the beta coefficients of the distance variables. Effect modification was explored as part of the multivariate model building process and did not yield any significant interactions. In addition to the unadjusted model two other models were developed, a model adjusted for variables that could influence exposure to air pollution (“exposure adjusted”) and a fully adjusted model that included the exposure variables. Residuals were checked and found to be normally distributed. We also fit generalized additive models (GAM) which allowed for a smooth effect of the continuous distance variables and generated corresponding spline plots for the 0-450 m study areas.

## Results

Participants were recruited between July 2009 and June 2011. Out of a random sample of 1,247 addresses, 587 were determined to be eligible and, of these, 327 (56%) completed surveys and 174 gave blood samples with one participant’s blood sample not viable for analysis (final N = 173). Ninety-four convenience participants are also included. In total we had blood samples from 267 people and used 260 of these for this analysis, eliminating 7 who lived outside the distance categories.

The mean age of participants was 58.2 years, 155 (58%) were women and most (66%) were White. The proportion of those who completed high school was 78%, most had incomes below $75,000 (69%) and mean BMI was 29.7. There was little difference with distance for near-highway population subgroups 0-50 m, 150-250 m and 250-450 m for age, BMI, household income, education, employment, study area, or traveling on highways (Table 1). It is important to note that the 50-150 m distance group was younger, had lower BMI, higher SES, and traveled less on highways, resembling the urban background population.

In the Somerville study area both hsCRP and IL-6 were higher in near highway areas than in the urban background (>1000 m), although a dose response relationship with distance was not apparent. Mean and median biomarker data by distance to highway for the total sample and by neighborhood (Additional file 1: Table S1). Fibrinogen and TNF-RII were not elevated near the highway in Somerville. Near highway levels were not elevated for any of the blood markers for the Dorchester area. There was little evidence of associations with distance in regression models for TNF-RII or fibrinogen (Additional file 2: Table S2).

**Figure 2:**
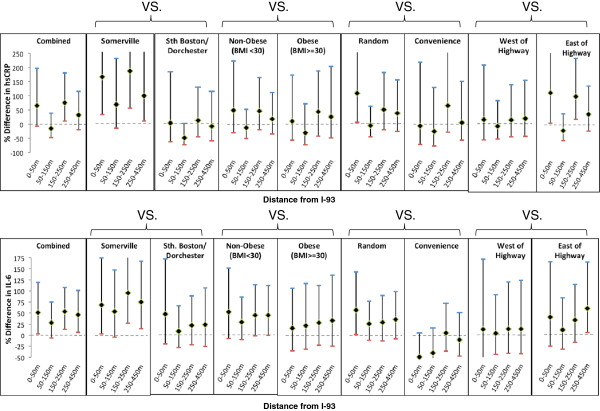
Unadjusted analysis of associations between distance and hsCRP and IL-6 levels, for various subpopulations compared to background.

In the unadjusted model hsCRP was higher near the highway compared to urban background except in the 50-150 m distance category (Table 2 and Figure 2). Adjustment for exposure modifiers resulted in a gradient from closer to farther from the highway, with the exception of 50-150 m residences. The fully adjusted model included age, smoking status, gender, income, BMI, born in the USA, vigorous physical activity, travel on highway, cooked with oil, non-workday time spent inside home, insulin medication, statin medication, heart attack. This model no longer had a distance-dependent gradient, although hsCRP remained elevated relative to urban background for all distance categories except 50-150 m.

**Table 2:** Regression models comparing hsCRP and IL-6 with distance from the highway

**Highway distance**	**Unadjusted model**		**Exposure adjusted**		**Adjusted model**	
	**(N = 260)**		**(N = 252)**		**(N = 225)**	
**hsCRP**	**%Diff**	**95%CI**	**%Diff**	**95%CI**	**%Diff**	**95%CI**
	**Adj. R**^**2**^ **= 0.05**		**Adj. R**^**2**^ **= 0.14**		**Adj R**^**2**^ **= 0.38**	
0-50 m	67%	(-8%,197%)	99%	(12%,254%)	43%	(-16%,141%)
50-150 m	-15%	(-48%,38%)	-24%	(-53%,22%)	7%	(-39%,45%)
150-250 m	75%	(9%,180%)	70%	(7%,169%)	54%	(-2%, 142%)
250-450 m	31%	(-20%,116%)	29%	(-27%,107%)	49%	(-4%,131%)
≥1000 m	ref	ref	ref			
**IL-6**	**Adj. R**^**2**^** = 0.04**		**Adj. R**^**2**^** = 0.17**		**Adj R**^**2**^** = 0.29**	
0-50 m	51%	(4%,119%)	72%	(20%,146%)	49%	(6%,110%)
50-150 m	28%	(-6%,75%)	29%	(-4%,73%)	41%	(6%,86%)
150-250 m	54%	(13%,108%)	43%	(7%,90%)	18%	(-11%, 57%)
250-450 m	46%	(-5%,101%)	50%	(11%,101%)	42%	(6%,89%)
≥1000 m	ref	ref	ref			

Values represent percent difference between distance category and urban background population.

Exposure adjusted models.

hsCRP adjusted for time spent at home, windows opened in winter and summer, smoking pack years and driving on highway.

IL 6 adjusted for time spent at home, windows opened in winter, work combustion exposures and air conditioner type.

Fully adjusted models.

hsCRP adjusted for age, smoking status, gender, income, BMI, born in the USA, vigorous physical activity, travel on highway, cooked with oil, non-workday time spent inside home, insulin medication, statin medication, heart attack.

IL6 adjusted for age, gender, smoking status, BMI, workday time spent at home, windows opened in winter and air conditioner type.

In the unadjusted model for IL-6, all of the near highway distance categories had positive associations relative to urban background (Table 2 and Figure 2). As with hsCRP, the exposure adjusted model increased the estimate for the < 50 m distance category. The fully adjusted model adjusted for age, gender, smoking status, BMI, workday time spent at home, windows opened in winter and air conditioner type. In this model all population groups had elevated IL-6 relative to urban background, though notably less for the 150-250 m population. In the fully adjusted models for hsCRP and IL-6 BMI was found to contribute the greatest amount to the adjusted R^2^ and was shown to be significantly associated with proximity to highway (results not shown).

Adjusted GAM models for the relationship between LN IL-6 and LN hsCRP and distance to highway in the 0-450 m study population (Additional file 3: Figure S1.) displayed a similar trend to the independent variable categorical distance. Stratification of adjusted GAM models by study area displayed markedly different patterns for LN hsCRP. Distance to highway was also examined as a continuous linear variable in adjusted models and while not significant had an inverse relationship with LN IL-6 and LN hsCRP (data not present here).

We also restricted the analysis for Table 2 to include only those participants with complete data for both hsCRP and IL-6 in the fully adjusted models (Additional file 4: Table S3, Additional file 5: Table S4). Percent differences in Additional file 4: Table S3 increased in the unadjusted models, but remained relatively similar to Table 2 in the exposure adjusted and fully adjusted models while standard errors widened in all models. Using the same restriction as for Table 3, Additional file 5: Table S4 compares geocoding methodologies adjusting for covariates, which reduced sample size further. Adjustment of variables revealed a quantitative shift in percent differences within each of the geocoding methods but the qualitative comparison between methods remained similar.

**Table 3:** Fully adjusted percent difference of biomarkers by geocoding methodology

	**Distance**	**Ortho Corrected**	**Parcel**	**StreetMap**	**TIGERLine**
		**% Diff (95%CI)**	**% Diff (95%CI)**	**% Diff (95%CI)**	**% Diff (95%CI)**
**HsCRP**	0-50 m	44% (-18%,151%)	49% (-12%,149%)	79% (-11%,258%)	51% (-25%,197%)
	51-150 m	-2% (-37%,53%)	2% (-34%,57%)	13% (-27%,73%)	22% (-21%,88%)
	151-250 m	40% (-11%,122%)	53% (-4%,143%)	22% (-25%,99%)	16% (-27%,85%)
	251-450 m	46% (-7%,128%)	46% (-7%,130%)	39% (-12%,119%)	57% (-33%,119%)
	> = 1000 m	Ref	Ref	Ref	Ref
**IL-6**	0-50 m	59% (2%,147%)	41% (-9%,119%)	27% (-37%,158%)	34% (-21%,128%)
	51-150 m	41% (-0.2%,100%)	45% (3%,105%)	18% (-21%,77%)	53% (10%,112%)
	151-250 m	4% (-27%,48%)	12% (-21%,60%)	-13% (-45%,38%)	3% (-27%,47%)
	251-450 m	60% (12%,128%)	55% (8%,123%)	8% (-31%,68%)	54% (5%,126%)
	> = 1000 m	Ref	Ref	Ref	Ref

The sample has been restricted to include those participants geocoded to all three methodologies and containing complete data for variables for each multi-variable regression model (Orthophoto and Parcel N = 223; TIGER N = 210).

We reran the unadjusted and adjusted hsCRP and IL-6 models using the parcel matched, StreetMap USA and TIGER address geocoding and found predominantly that there were changes in associations toward the null for the StreetMap USA and TIGER addresses. The effect of geocoding error on directionality of effect for model ß-estimates was not systematic. The confidence intervals (95%) changed in non-uniform ways, resulting in some spurious results (Table 3 & Additional file 5: Table S4). Distance bin misclassification was examined for the TIGER and Parcel geocoding methodologies by comparing to the ortho-photo corrected residential locations. TIGER geocoding had more false negatives and less sensitivity than parcel geocoding in all distance bins (Additional file 6: Table S5).

We examined medications in detail. Statins, OHAs, and antihypertensives were associated with higher levels of all biomarkers in crude associations. Antacid use was associated with higher levels of hsCRP, IL-6 and TNF-RII. Anti-inflammatory medications and hormones were not associated with differences in biomarkers. In regression models, inclusion of BMI often resulted in medications losing significance. When BMI was excluded from models, some medications could be included; however, this was usually antihypertensive treatment, acting in the same direction as BMI, and likely collinear with BMI in the models (Additional file 7: Table S6). Overall, we found that medications had nominal impact on associations and were included in only two of the models in Table 2.

We also examined reported combustion exposures in the week preceding the blood draw. In adjusted regression models several exposures were associated with cooking with oil for hsCRP and IL-6; spending time on a city street for 20 minutes for IL-6 (in the opposite direction from expected; Additional file 8: Table S7), and smoke exposure at work for TNF-RII (results not shown). Of these, only cooking with oil made it into our fully adjusted model for hsCRP (Table 2). Cooking with oils generates UFP, but we were not able to distinguish effects of food consumption from inhalation of aerosolized oil and found no literature that addressed this issue [19].

**Figure 3:**
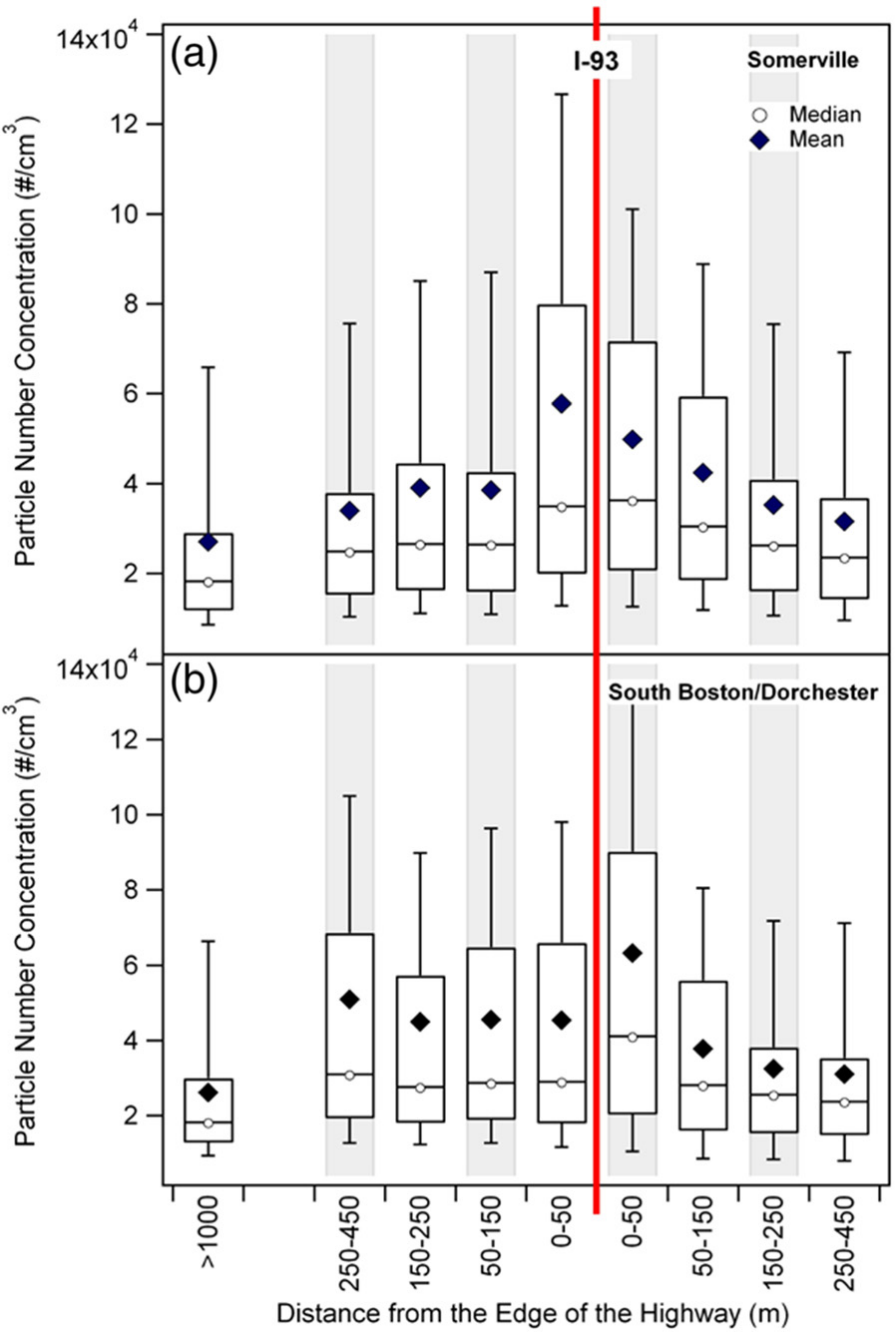
**Box plots of 1-second PNC measurements as a function of distance from I-93 for Somerville (a) and Dorchester/South Boston (b).** The boxes represent the 25^th^ and 75^th^ percentiles of the data, the whiskers represent the 10^th^ and 90^th^ percentiles. The horizontal solid line in each box represents the median PNC; the black diamond represents the average PNC. The right side of the red line indicates distance to the east of I-93 (generally downwind), and the left side indicates distance to the west of I-93 (generally upwind).

To inform subgroup analyses and interpret proximity measures, we compared proximity associations to box plots of UFP concentrations from mobile monitoring in Somerville and Dorchester (Figure 3). UFP were elevated on both sides of the highway in Somerville and for the east side (right side of figure) in Dorchester. The west side (left side of figure, predominantly upwind and with higher local traffic loads) of the highway in Dorchester had a flatter pattern with less evidence of elevation next to the highway (Figure 3b). A prominent sound wall along the east edge of I-93 in Somerville may also have affected concentrations. Concentrations were skewed to the right (approximately lognormal, outliers not shown). For each study area, mean and median UFP concentrations <450 m from the highway were higher than the same statistics in the urban background.

In subgroup analyses of unadjusted hsCRP and IL-6 (Figure 2) we found that associations were stronger in Somerville, in non-obese participants (particularly for IL-6) and in the random sample compared to the convenience sample. Associations were also stronger (especially for hsCRP) on the east side of the highway, which is predominantly downwind. We found less consistent differences in associations by native vs. foreign born, age, gender and smoking or diabetes status (Additional file 9: Figure S2 and Figure S3).

## Discussion

Using precise geo-positioning for residential addresses and screening a large number of potential confounders we found associations of distance to highway with hsCRP and IL-6. However, we found little evidence for associations for TNF-RII or fibrinogen with proximity. Associations of hsCRP and IL-6 with each other and with cardiovascular disease (CVD) are well established in the literature. The risk ratio for coronary heart disease for a 3-fold higher hsCRP level in a large meta analysis was 1.63, suggesting that if our associations were shown to be causal they could have an impact on morbidity and mortality for near highway residents [20].

Previous research has shown that geocoding addresses to street networks results in substantial misclassification for proximity studies requiring a high degree of spatial accuracy [13,15,21]. Our results expand upon these findings and indicate that misclassification can result in biased regression models (Table 3 and Additional file 5: Table S4). Misclassification was differential in our data set, as those closest to the highway had the greatest classification error, attributable in part to street network geocoding [15]. Studies that require fine-scale spatial resolution such as a near highway analysis should, at a minimum, use local parcel data for geocoding in order to limit the effects of positional error and should consider ortho-photo matching.

Geocoding to tax parcel databases has been used less frequently, but has been shown in this study and others to introduce less positional error than geocoding to street networks [12,14]. Parcel datasets are primarily created at the city or county planning level. It requires collaboration with city planners to gain access to these geo-databases. Ortho-photo imagery is readily available through ESRI ArcGIS, but temporal accuracy and spatial resolution may vary across different areas. We were fortunate that MassGIS has compiled statewide parcel and ortho-photo datsets and made them freely available to download from a single website easing the process of obtaining these datsets in Massachusetts. Researchers working with large cohorts will need to weigh the benefits of reducing positional error against the additional computational resources and time requirements of ortho-photo correction. However, the increase in exposure accuracy can be considerable.

We back calculated expected effect sizes from the literature to test the consistency of our findings with those of others. Because none of the studies comparing highway proximity and hsCRP had data that could be compared to ours, we started with Panasevich et al. who found a statistically significant correlation between long-term exposure to elevated residential NO_2_ and higher hsCRP and IL-6 (5-year exposure values from Table 2 of their publication) [22]. Since NO_2_ concentrations decay next to highways, we used the NO_2_ distance-decay slope for a highway similar to that of our study area, calculated by Gilbert et al. (linear regression model with the highest R^2^), to convert NO_2_ levels from Panasevich to distance [23]. Using these two studies, we estimated that hsCRP and IL-6 levels within 100 m of the highway might be expected to be 11% and 24% higher, respectively, than for those living further than 1000 m away. The actual effect sizes we found were mostly 2–5 times higher (Table 2). One possible source of difference, other than study methodology and differences in location, could be that NO_2_ gradients decay more gradually than do UFP gradients next to highways, and that UFP is more likely to be the causal agent [8]. Another possibility is that we had a vulnerable population with high prevalence of obesity and diabetes relative to the comparison study. Still, our estimates of effect appear higher than previous estimates in general, especially for the random sample and for the Somerville subset.

UFP decay patterns were similar to the relationship between hsCRP and IL-6 using categorical distance to highway. The biomarker associations we found for distance from the highway were relatively flat across distance categories, except for the 50-150 m category for hsCRP. Associations of hsCRP and IL-6 with distance were lower on the west side of the highway (Figure 2), where UFP concentrations were lower and gradients were less pronounced (Figure 3). UFP gradients in both neighborhoods were steeper east of the highway (usually downwind; right side of Figure 3) than west, perhaps due to busy local roadways and wind direction. In a detailed analysis reported elsewhere, this UFP difference between west (upwind) and east (downwind) highway sides held for analysis by categories including season, time of day, day of week, wind speed and wind direction [18]. These factors may account in part for the substantial differences in distance associations for hsCRP and IL-6 between Somerville and Dorchester. In particular local street traffic may contribute to UFP exposures especially in the urban background area in Dorchester where participants resided much closer to a major roadway.

In our analysis of hsCRP, the 50-150 m distance category was anomalous and did not have elevated levels relative to background, even in the fully adjusted models. The population living 50-150 m from the highway was demographically similar to the population in the comparison group (urban background). As Table 1 clearly shows, there are appreciable individual level socioeconomic differences between populations in different distance categories. While there was an indication of a smaller but similar pattern in IL-6 models, the fully adjusted model brought the 50-150 m category in line with other near highway categories, suggesting confounding. IL-6 promotes the release of hsCRP, so it is not surprising that we found similar responses. But we cannot explain why controlling for confounding did not adjust the 50-150 m hsCRP associations as it did for IL-6. Adjusting for potential confounders failed to eliminate the possibility of residual confounding based on the results for hsCRP in the 50-150 m group.

### Limitations and strengths

Our sample size was modest and there was considerable heterogeneity of the populations in distance categories (Additional file 2: Table S2), which increased the risk of residual confounding. Despite our random sample, our analysis may have limited generalizability. Indications of limitation include the difference in findings between our study areas, the exclusion of one study area due to geographic complexity and between the random and convenience samples. If such variability in response exists within our sample, it is likely that our sample and other populations also will vary. Additionally, we would expect our population to be better matched with populations in the Northeastern US than in other parts of the country or the world.

Our primary exposure metric, distance from the highway, likely introduced exposure misclassification relative to what might be seen with individually-assigned exposures to UFP. We also did not test associations with traffic or topographic metrics other than distance to the highway. We have shown elsewhere [15] that for near highway residents misclassification was differential for time spent away from home, which could reduce exposure. Controlling for time activity and other exposure modifiers enhanced near-highway associations.

A particular strength of this analysis was the use of precise geocoding for residential addresses, achieving the “gold standard” in the field. We recruited in 6 languages, increasing our sampling of hard to reach residents. Our sample was stratified by distance from the highway to maximize exposure contrast. We screened for a large number of potential confounders which included many variables not usually assessed in highway proximity studies, however, we could not assess the impact of traffic or other ambient noise. We explored in full regression models the role of medications and other sources of exposure to combustion. We also had measurements of UFP from the study areas from the same year in which we recruited participants and made a separate qualitative comparison of UFP gradients with associations of distance with hsCRP and IL-6. Finally, we had objective measures of both distance and health.

## Conclusion

Our results suggest that highway proximity affects blood markers of inflammation which are, in turn, associated with increased cardiovascular disease risk. Highway proximity is associated with UFP and other pollutants, but also SES and traffic noise. We point to three main lessons from this analysis: 1) Attention to high standards in geocoding is valuable, as less rigorous approaches led to different results; 2) Individual level confounding is a threat to valid associations; and 3) Side of highway and predominant wind direction affected associations, emphasizing limitations in proximity measures. By addressing these issues, we feel that we have improved confidence that traffic pollution next to highways is a risk factor for cardiovascular disease. Future research will need to go beyond using proximity and, instead, assign individual exposures to residents, ideally moving toward personal exposure measures that would decrease potential confounding due to other distance-dependent factors.

## Abbreviations

ArcGIS: Geographic Information System Software; CAFEH: Community Assessment of Freeway Exposure and Health; CO: Carbon Monoxide; CVD: Cardiovascular Disease; GIS: Geographic Information System; hsCRP: High-Sensitivity C-reactive Protein; IL-6: Interleukin-6; NO: Nitric oxide; NOx: Nitrogen oxides; NO2: Nitrogen Dioxide; Orthoimage: Aerial photograph that is geometrically corrected; SES: Socioeconomic Status; TAPL: Tufts Mobile Air Pollution Laboratory; TIGER: Topologically Integrated Geographic Encoding and Referencing; TNF-RII: Tumor necrosis factor receptor type 2; UFP: Ultrafine Particles.

## Competing interests

Brugge has received travel support to make presentations about uranium mining from Friends of the Earth and International Physicians for the Prevention of Nuclear War. Funding was provided by the National Institute of Environmental Health Sciences (ES015462). Padró-Martínez and Brugge were also supported by HUD grant MALHH0194-09. Support for Lane and Patton was provided by EPA STAR Fellowships (FP-917349-01-0; FP-91720301-0).

## Authors’ contributions

DB led and directed the study, provided oversight to the analysis and was the lead writer. KL was the lead analyst and contributed to writing, LTP-M led the analysis of the air monitoring data, AS contributed to the literature review and did the analysis presented in the discussion, KH did the medication analysis, DW did the combustion exposure analysis, DDW contributed to the analysis, JIL contributed meaningful intellectual ideas during the writing that affected the interpretation of our data, APP contributed to the air pollution analysis, WZ helped initiate and design the overall study and contributed intellectually to its interpretation, MM oversaw the statistical analysis and wrote that section of the paper. All authors read the manuscript multiple times, provided input and approved the version as submitted.

## Supplementary Material

Additional file 1: Table S1 Mean and median values for blood biomarkers stratified by distance to the highway. Click here for file

Additional file 2: Table S2 Regression models comparing fibrinogen and TNF-RII with distance from the highway. Values for fibrinogen represent absolute differences (mg/dl) between distance category and urban background population, and values for TNF-RII represent percent differences between distance category and urban background population. Click here for file

Additional file 3: Figure S1 LOESS smooth plots of predicted LN IL-6 and LN hsCRP from Fully Adjusted Generalized Additive Models. Click here for file

Additional file 4: Table S3 Regression models comparing hsCRP and IL-6 with distance from the highway for orthophoto corrected geocoded residential positions. Values represent percent difference between distance category and urban background population restricted to include those participants containing complete data for all variables in the fully adjusted multi-variable regression models for LN of hsCRP and IL-6 (N = 223). Click here for file

Additional file 5: Table S4 Unadjusted percent difference of biomarkers by geocoding methodology. This table has different sample sizes from Table 1 due to participants in the 450-1000 m groups being removed from the analysis. Click here for file

Additional file 6: Table S5 Distance bin misclassification by geocoding methodology. The analysis includes individuals in the 450-1000 m distance group to provide exhaustive distance coverage but omits those not successfully geocoded to both the TIGERline and Parcel datasets (n = 262). Confirmed match represents the number of residences classified in the distance group by each geocoding method and orthophoto corrected location assignment.% False negatives indicate the number of residences that should have been in the distance bin but were geocoded to an incorrect bin divided by the total sample size (n = 262).% False positives indicate the number of residences that were incorrectly geocoded to the distance bin divided by the total sample size (n = 262). Sensitivity is the percentage of confirmed positive residences for each distance bin (confirmed match divided by orthophoto corrected). Specificity is the percentage of correctly identified negative residences for each distance bin. Click here for file

Additional file 7: Table S6 Regression models of medication usage by group. Values represent percent differences between individuals taking versus not taking the listed medications. Click here for file

Additional file 8: Table S7 Regression models of combustion exposure in the 2 weeks preceding the blood draw by group. Values represent percent differences between individuals with and without the exposure. Click here for file

Additional file 9: Figure S2 Unadjusted analysis of associations between distance and hsCRP and IL-6 levels by age, Born USA and Smoking. **Figure S3** Unadjusted analysis of associations between distance and hsCRP and IL-6 levels by gender and diabetic. Click here for file

## References

[B1] Tonne C Melly S Mittleman M Coull B Goldberg R Schwartz J A case–control analysis of exposure to traffic and acute myocardial infarction Environ Health Perspect 2007 12 53 57 17366819 10.1289/ehp.9587 PMC1797833

[B2] Gan WQ Tamburic L Davies HW Demers PA Koehoorn M Brauer M Changes in residential proximity to road traffic and the risk of death from coronary heart disease Epidemiology 2010 12 642 649 10.1097/EDE.0b013e3181e89f19 20585255

[B3] Hoffmann B Moebus S Möhlenkamp S Stang A Lehmann N Dragano N Schmermund A Memmesheimer M Mann K Erbel R Jöckel K Residential exposure to traffic is associated with coronary atherosclerosis Heinz Nixdorf Recall Study Invest Group 2007 12 489 496 10.1161/CIRCULATIONAHA.107.693622 17638927

[B4] Dubowsky SD Suh H Schwartz J Coull BA Gold DR Diabetes, obesity, and hypertension may enhance associations between air pollution and markers of systemic inflammation Environ Health Perspect 2006 12 992 998 10.1289/ehp.8469 16835049 PMC1513328

[B5] Rioux CL Tucker KL Mwamburi M Gute DM Cohen SA Brugge D Residential traffic exposure, pulse pressure, and C-reactive protein: consistency and contrast among exposure characterization methods Environ Health Perspect 2010 12 803 811 10.1289/ehp.0901182 20123638 PMC2898857

[B6] Williams LA Ulrich CM Larson T Wener MH Wood B Campbell P Potter J McTiernan A De Roos A Proximity to traffic, inflammation, and immune function among women in the Seattle, Washington, area Environ Health Perspect 2009 12 374 378 10.1289/ehp.11580 PMC2661906 19337511

[B7] Hoffmann B Moebus S Dragano N Stang A Möhlenkamp S Schmermund A Memmesheirmer M Brocker-Preuss M Mann K Erbel R Jockel K Chronic residential exposure to particulate matter air pollution and systemic inflammatory markers Environ Health Perspect 2009 12 1302 1308 19672412 10.1289/ehp.0800362 PMC2721876

[B8] Brugge D Durant JL Rioux C Near-highway pollutants in motor vehicle exhaust: a review of epidemiologic evidence of cardiac and pulmonary health risks Environ Health 2007 12 23 10.1186/1476-069X-6-23 17688699 PMC1971259

[B9] Karner AA Eisinger DS Niemeier DA Near-roadway air quality: synthesizing the findings from real-world data Environ Sci Technol 2010 12 5334 5344 10.1021/es100008x 20560612

[B10] Durant JL Ash CA Wood EC Herndon SC Jayne JT Knighton WB Canagaratna MR Trull JB Brugge D Zamore W Kolb CE Short-term variation in near-highway air pollutant gradients on a winter morning Atmos Chem Phys 2010 12 8341 8352 10.5194/acpd-10-5599-2010 PMC3304588 22427751

[B11] Babisch W Transportation noise and cardiovascular risk: updated review and synthesis of epidemiological studies indicate that evidence has increased Noise Health 2006 12 1 29 10.4103/1463-1741.32464 17513892

[B12] Cayo MR Talbot TO Positional error in automated geocoding of residential addresses Int J Health Geogr 2003 12 10 10.1186/1476-072X-2-10 14687425 PMC324564

[B13] Zandbergen P Green J Error and bias in determining exposure potential of children at school locations using proximity-based GIS techniques Environ Health Perspect 2007 12 1363 1370 10.1289/ehp.9668 17805429 PMC1964899

[B14] Schootman M Sterling D Strutters J Yan Y Laboube T EMO B Higgs G Positional accuracy and geographic bias of four methods of geocoding in epidemiologic research Ann Epidemiol 2007 12 464 470 10.1016/j.annepidem.2006.10.015 17448683

[B15] Lane KJ Scammell MK Levy JI Fuller CH Parambi R Zamore W Mwamburi M Brugge D Exposure misclassification related to positional error and time-activity patterns in studies of near-highway health effects Environmental Health 2013 12 75 10.1186/1476-069X-12-75 24010639 PMC3907019

[B16] Fuller CH Patton AP Lane K Laws MB Marden A Carrasco E Spengler J Mwamburi M Zamore W Durant JL Brugge DA Community participatory study of cardiovascular health and exposure to near-highway air pollution: study design and methods Rev Environ Health 2013 12 1 72 23612527 10.1515/reveh-2012-0029 PMC3708485

[B17] MassGIS (Massachusetts Office of Geographic Information) USGS color ortho imagery (2008/2009) 2008 http://www.mass.gov/anf/research-and-tech/it-serv-and-support/application-serv/office-of-geographic-information-massgis/datalayers/colororthos2008.html

[B18] Padró-Martínez LT Patton A Trull JB Zamore W Brugge D Durant JL Mobile monitoring of spatial and temporal variation of traffic-related air pollution in a near-highway urban neighborhood over the course of a year Atmos Environ 2012 12 253 264 10.1016/j.atmosenv.2012.06.088 PMC3491988 23144586

[B19] Torkmahalleh MA Goldasteh I Zhao Y Udochu NM Rossner A Hopke PK Ferro AR PM_2.5_ and ultrafine particles emitted during heating of commercial cooking oils Indoor Aironline in advance of print 2012, doi:1111/j.1600-0668.2012.00783.x 10.1111/j.1600-0668.2012.00783.x 22486983

[B20] The Emerging Risk Factors Collaboration C-reactive protein concentration and risk of coronary heart disease, stroke, and mortality: an individual participant meta-analysis Lancet 2010 12 132 140 20031199 10.1016/S0140-6736(09)61717-7 PMC3162187

[B21] Zandbergen P Influence of geocoding quality on environmental exposure assessment of children living near high traffic roads BMC Public Health 2007 12 37 10.1186/1471-2458-7-37 17367533 PMC1838415

[B22] Panasevich S Leander K Rosenlund M Ljungman P Bellander T De Faire U Pershagen G Nyberg F Associations of long-and short-term air pollution exposure with markers of inflammation and coagulation in a population sample Occup Environ Med 2009 12 747 753 10.1136/oem.2008.043471 19687019

[B23] Gilbert NL Woodhouse S Stieb DM Brook JR Ambient nitrogen dioxide and distance from a major highway Sci Total Environ 2003 12 43 46 10.1016/S0048-9697(03)00228-6 12873397

