# Supplementary material for: Highway proximity associated with cardiovascular disease risk: the influence of individual-level confounders and exposure misclassification
Source: Environ Health. 2013 Oct 3;12:84. doi: 10.1186/1476-069X-12-84 (PMC3907023; doi:10.1186/1476-069X-12-84)
Supplement: Additional file 1: Table S1 — Mean and median values for blood biomarkers stratified by distance to the highway. [file 1476-069X-12-84-S1.pdf]

Supplemental Table 1. Mean and median values for blood biomarkers stratified by distance to the highway.

Biomarker	Within 50m		50 – 150m		150 – 250m		250 – 450m		>1000m	
	(N=30)		*(N=58)*		*(N=65)*		*(N=54)*		*(N=53)*	
	Mean(SD)	Median	Mean(SD)	Median	Mean(SD)	Median	Mean(SD)	Median	Mean(SD)	Median
**hsCRP(mg/dl)**										
Combined	5.34(8.76)	1.79	3.38(8.4)	1.21	6.67(12.24)	2.13	3.55(4.23)	2.35	3.53(6.88)	1.36
Somerville	5.19(7.74)	1.92	4.45(12.18)	1.53	7.84(14.93)	2.02	3.37(4.06)	2.18	1.6(2.07)	0.94
S. Boston/ Dorchester	5.66(10.96)	1.35	2.73(4.99)	0.98	5.36(8.27)	2.4	3.95(4.68)	2.94	5.39(9.12)	2.15
**IL-6(pg/ml)**										
Combined	2.91(2.77)	1.63	2.26(2.14)	1.58	3.27(6.04)	1.79	3.03(4.15)	1.74	1.84(1.8)	1.27
Somerville	2.74(2.68)	1.63	2.38(2.45)	1.77	3.84(7.8)	1.95	2.75(3.62)	1.66	1.36(1.01)	0.97
S. Boston/ Dorchester	3.26(3.06)	2.36	2.19(1.95)	1.41	2.59(2.87)	1.68	3.62(5.17)	1.75	2.28(2.23)	1.47
**Fibrinogen(mg/dl)**										
Combined	387(113)	384	366(96)	349	4.16(97)	399	391(82)	387	387(105)	362
Somerville	366(120)	351	373(91)	372	421(94)	404	392(83)	385	349(75)	332
S. Boston/ Dorchester	428(88)	401	362(100)	338	409(100)	382	388(83)	390	423(117)	422
**TNF-RII(pg/ml)**										
Combined	2683(999)	2534	2570(1231)	2245	2949(1439)	2501	3084(1905)	2595	2658(1339)	2349
Somerville	2660(817)	2574	2802(1525)	2270	3543(1590)	3013	3162(1366)	2855	2463(810)	2299
S. Boston/ Dorchester	2732(1345)	2302	2425(1003)	2155	2258(829)	2013	2915(2789)	2292	2853(1709)	2363
