# Supplementary material for: Highway proximity associated with cardiovascular disease risk: the influence of individual-level confounders and exposure misclassification
Source: Environ Health. 2013 Oct 3;12:84. doi: 10.1186/1476-069X-12-84 (PMC3907023; doi:10.1186/1476-069X-12-84)
Supplement: Additional file 2: Table S2 — Regression models comparing fibrinogen and TNF-RII with distance from the highway. Values for fibrinogen represent absolute differences (mg/dl) between distance category and urban background population, and values for TNF-RII represent percent differences between distance category and urban background population. [file 1476-069X-12-84-S2.pdf]

Supplemental Table 2. Regression models comparing fibrinogen and TNF-RII with distance from the highway. Values for fibrinogen represent absolute differences (mg/dl) between distance category and urban background population, and values for TNF-RII represent percent differences between distance category and urban background population.

Highway Distance	Unadjusted Model		Exposure Adjusted		Adjusted Model	
	(N=260)		(N=240)		(N=231)	
Fibrinogen (mg/dl)	Abs. Diff	95%CI	Abs. Diff	95%CI	Abs. Diff	95%CI
	Adj R2 = 0.03		Adj R2 = 0.15		Adj R2 = 0.35	
0-50m	0.11	(-43.7,43.9)	51.09	(-0.21,102.4)	-4.46	(-43.4,34.5)
50-150m	-20.49	(-56.9,15.9)	0.49	(-39.7,40.7)	-4.53	(-36.7,27.6)
150-250m	28.83	(-6.5,64.2)	45.61	(6.3,84.9)	10.51	(-22.4,43.4)
250-450m	4.24	(-32.8,41.3)	25.29	(-17.5,68.1)	0.72	(-31.7,33.11)
≥1000m	*ref*		*ref*		*ref*	
**Highway Distance**	**Unadjusted Model**		**Exposure Adjusted**		**Adjusted Model**	
	(N=257)		(N=240)		(N=225)	
TNF-RII	% Diff	95%CI	% Diff	95%CI	% Diff	95%CI
	Adj R2 = 0.02		Adj R2 = 0.12		Adj R2 = 0.33	
0-50m	4%	(-14%,24%)	-1%	(-28%,20%)	-9%	(-24%,9%)
50-150m	-3%	(-16%,13%)	-7%	(-20%,7%)	-8%	(-20%,7%)
150-250m	10%	(-5%,27%)	1%	(-12%,17%)	-9%	(-21%,5%)
250-450m	13%	(3%,31%)	-2%	(-17%,14%)	-1%	(-15%,14%)
≥1000m	*ref*		*ref*		*ref*	

Exposure model

Fibrinogen adjusted for total time spent at home, windows opened in summer, pack years, residence side of highway, distance to major roadway and combustion score

Fully adjusted model

Fibrinogen adjusted for age, gender, BMI, smoking status, previous heart attack, statin medication, insulin medication, vigorous physical activity and income.

Exposure model

TNF-RII adjusted for total time spent at home, total time spent on highways, distance to major roadways, pack years and other combustion source score.

Fully adjusted model

TNF-RII adjusted for age, gender, BMI, smoking status, congestive heart failure, distance to major roadway, white/nonwhite, vigorous physical activity and educational attainment.
