# Supplementary material for: Highway proximity associated with cardiovascular disease risk: the influence of individual-level confounders and exposure misclassification
Source: Environ Health. 2013 Oct 3;12:84. doi: 10.1186/1476-069X-12-84 (PMC3907023; doi:10.1186/1476-069X-12-84)

Supplemental Figure 1. LOESS smooth plots of predicted LN IL-6 and LN hsCRP from Fully Adjusted Generalized Additive Models.

LN IL-6

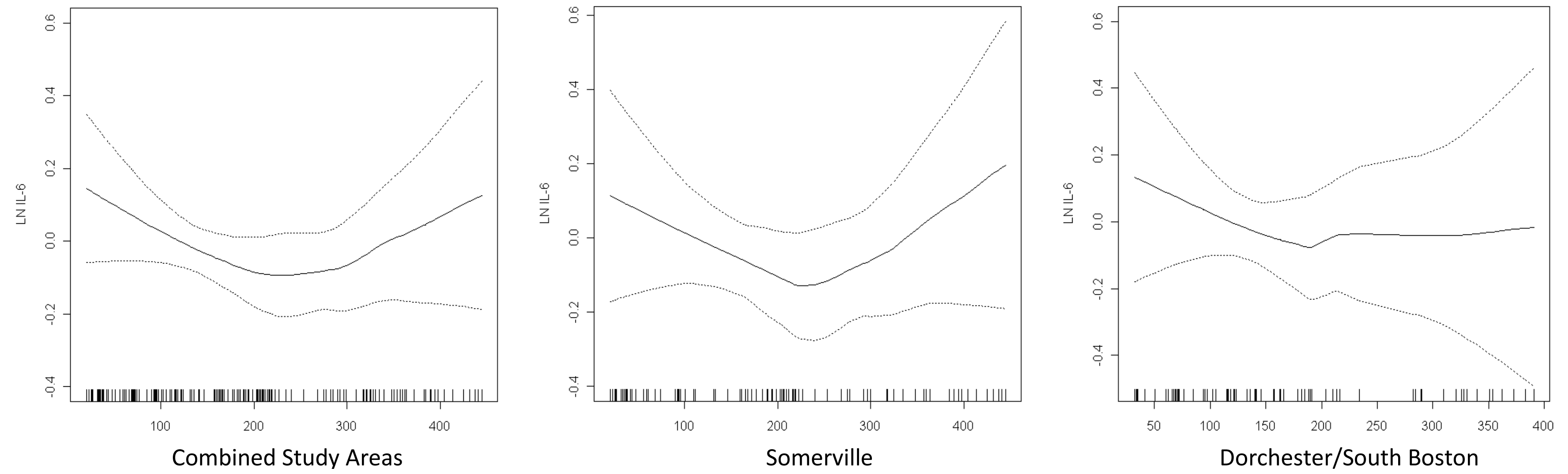

LN hsCRP

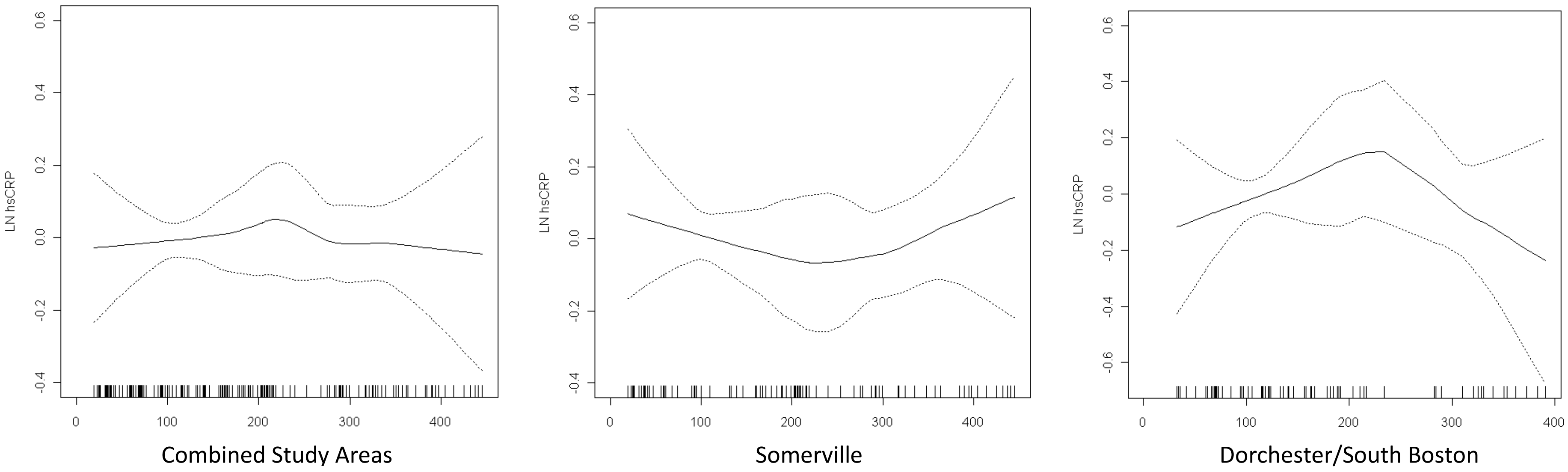

Supplement: Additional file 3: Figure S1 — LOESS smooth plots of predicted LN IL-6 and LN hsCRP from Fully Adjusted Generalized Additive Models. [file 1476-069X-12-84-S3.pdf]
