# Supplementary material for: Highway proximity associated with cardiovascular disease risk: the influence of individual-level confounders and exposure misclassification
Source: Environ Health. 2013 Oct 3;12:84. doi: 10.1186/1476-069X-12-84 (PMC3907023; doi:10.1186/1476-069X-12-84)
Supplement: Additional file 4: Table S3 — Regression models comparing hsCRP and IL-6 with distance from the highway for orthophoto corrected geocoded residential positions. Values represent percent difference between distance category and urban background population restricted to include those participants containing complete data for all variables in the fully adjusted multi-variable regression models for LN of hsCRP and IL-6 (N = 223). [file 1476-069X-12-84-S4.pdf]

Supplemental Table 3. Regression models comparing hsCRP and IL-6 with distance from the highway for orthophoto corrected geocoded residential positions. Values represent percent difference between distance category and urban background population restricted to include those participants containing complete data for all variables in the fully adjusted multi-variable regression models for LN of hsCRP and IL-6 (N=223).

Highway Distance	*Unadjusted Model*		*Exposure Adjusted*		*Adjusted Model*	
hsCRP	%Diff	95%CI	%Diff	95%CI	%Diff	95%CI
	Adj. R^2=0.07		Adj. R^2=0.12		Adj R^2=0.40	
0-50m	100%	(5%,280%)	98%	(5%,280%)	44%	(-18%,151%)
50-150m	-8%	(-45%,43%)	-17%	(-45%,53%)	-2%	(-37%,53%)
150-250m	101%	(21%,231%)	67%	(-22%,231%)	40%	(-11%,122%)
250-450m	65%	(-2%,178%)	46%	(-2%,178%)	46%	(-7%,128%)
≥1000m	*ref*		*ref*		*ref*	
IL-6	Adj. R^2=0.05		Adj. R^2=0.20		Adj R^2=0.32	
0-50m	74%	(6%,187%)	96%	(22%,214%)	59%	(22%,213%)
50-150m	52%	(3%,25%)	37%	(-6%,99%)	41%	(-0.2%,99%)
150-250m	60%	(8%,35%)	28%	(-12%,86%)	4%	(-27%,86%)
250-450m	99%	(33%,200%)	77%	(20%,160%)	60%	(12%,160%)
≥1000m	*ref*		*ref*		*ref*	

Exposure adjusted models

hsCRP adjusted for time spent at home, windows opened in winter and summer, smoking pack years and driving on highway
IL 6 adjusted for time spent at home, windows opened in winter, work combustion exposures and air conditioner type

Fully adjusted models

hsCRP adjusted for age, smoking status, gender, income, BMI, born in the USA, vigorous physical activity, travel on highway, cooked with oil, non-workday time spent inside home, insulin medication, statin medication, heart attack.
IL6 adjusted for age, gender, smoking status, BMI, workday time spent at home, windows opened in winter and air conditioner type
