# Supplementary material for: Highway proximity associated with cardiovascular disease risk: the influence of individual-level confounders and exposure misclassification
Source: Environ Health. 2013 Oct 3;12:84. doi: 10.1186/1476-069X-12-84 (PMC3907023; doi:10.1186/1476-069X-12-84)
Supplement: Additional file 5: Table S4 — Unadjusted percent difference of biomarkers by geocoding methodology. This table has different sample sizes from Table 1 due to participants in the 450-1000 m groups being removed from the analysis. [file 1476-069X-12-84-S5.pdf]

Supplemental Table 4. Unadjusted percent difference of biomarkers by geocoding methodology. This table has different sample

	Distance	Ortho Corrected<br>N= 260<br>% Diff (95%CI)	Parcel<br>N= 252<br>% Diff (95%CI)	StreetMap<br>N= 246<br>% Diff (95%CI)	TIGERLine<br>N= 240<br>% Diff (95%CI)
HsCRP	0-50m	67% (-8%,197%)	73% (10%,235)	46% (-34%,27%)	37% (-37%,99%)
	51-150m	-15% (-48%,38%)	-7% (-42%,50%)	10% (-38%,56%)	19% (-26%,90%)
	151-250m	75% (9%,180%)	87% (17%,198%)	24% (-27%,75%)	51% (-6%,143%)
	251-450m	31% (-20%,116%)	30% (-22,114)	36% (-13%,86%)	24% (-29,115%)
	>=1000m	Ref	Ref	Ref	Ref
IL-6	0-50m	51 (4%:119%)	36% (-11%:108)	6% (-64%,75%)	22% (-25%:98%)
	51-150m	28% (-6%:75%)	29% (-5%:76%)	21% (-19%,61%)	47% (9%:97%)
	151-250m	54% (13%:108%)	50% (11%:103%)	14% (-30%,58%)	52% (13%:106%)
	251-450m	46% (6%:101%)	35% (-3%:86%)	26% (-16%,69%)	24% (-11%:76%)
	>=1000m	Ref	Ref	Ref	Ref

sizes from Table 1 due to participants in the 450-1000 m groups being removed from the analysis.
