# Supplementary material for: Highway proximity associated with cardiovascular disease risk: the influence of individual-level confounders and exposure misclassification
Source: Environ Health. 2013 Oct 3;12:84. doi: 10.1186/1476-069X-12-84 (PMC3907023; doi:10.1186/1476-069X-12-84)
Supplement: Additional file 6: Table S5 — Distance bin misclassification by geocoding methodology. The analysis includes individuals in the 450-1000 m distance group to provide exhaustive distance coverage but omits those not successfully geocoded to both the TIGERline and Parcel datasets (n = 262). Confirmed match represents the number of residences classified in the distance group by each geocoding method and orthophoto corrected location assignment.% False negatives indicate the number of residences that should have been in the distance bin but were geocoded to an incorrect bin divided by the total sample size (n = 262).% False positives indicate the number of residences that were incorrectly geocoded to the distance bin divided by the total sample size (n = 262). Sensitivity is the percentage of confirmed positive residences for each distance bin (confirmed match divided by orthophoto corrected). Specificity is the percentage of correctly identified negative residences for each distance bin. [file 1476-069X-12-84-S6.pdf]

Supplemental Table 5. Distance bin misclassification by geocoding methodology. The analysis includes individuals in the 450-1000m distance group to provide exhaustive distance coverage but omits those not successfully geocoded to both the TIGERline and Parcel datasets (n=262)

Distance Group (m)	# of Residences by Proximity to Highway			Measures of Agreement			
	**Orthophoto Corrected**	**TIGER Geocode**	**Confirmed Match**	**% False Negatives**	**% False Positives**	**Sensitivity**	**Specificity**
0-50	27	14	13	5.34%	0.38%	48.15%	99.57%
50-150	57	69	49	3.05%	7.63%	85.96%	90.24%
150-250	65	65	57	3.05%	3.05%	87.69%	95.94%
250-450	54	37	36	6.87%	0.38%	66.67%	99.52%
450-1000	6	22	5	0.38%	6.49%	83.33%	93.36%
>=1000	53	55	53	0.00%	0.76%	100.00%	99.04%
	**Orthophoto Corrected**	**Parcel Geocode**	**Confirmed Match**	**% False Negatives**	**% False Positives**	**Sensitivity**	**Specificity**
0-50	27	21	21	2.29%	0.00%	77.78%	100.00%
50-150	57	63	57	0.00%	2.29%	100.00%	97.07%
150-250	65	67	64	0.38%	1.15%	98.46%	98.48%
250-450	54	50	50	1.53%	0.00%	92.59%	100.00%
450-1000	6	6	6	0.00%	0.76%	100.00%	99.22%
>=1000	53	55	53	0.00%	0.00%	100.00%	100.00%

Confirmed match represents the number of residences classified in the distance group by each geocoding method and orthophoto corrected location assignment.

% False negatives indicate the number of residences that should have been in the distance bin but were geocoded to an incorrect bin divided by the total sample size (n=262).

% False positives indicate the number of residences that were incorrectly geocoded to the distance bin divided by the total sample size (n=262).

Sensitivity is the percentage of confirmed positive residences for each distance bin (confirmed match divided by orthophoto corrected).

Specificity is the percentage of correctly identified negative residences for each distance bin.
