# Supplementary material for: Highway proximity associated with cardiovascular disease risk: the influence of individual-level confounders and exposure misclassification
Source: Environ Health. 2013 Oct 3;12:84. doi: 10.1186/1476-069X-12-84 (PMC3907023; doi:10.1186/1476-069X-12-84)
Supplement: Additional file 7: Table S6 — Regression models of medication usage by group. Values represent percent differences between individuals taking versus not taking the listed medications. [file 1476-069X-12-84-S7.pdf]

Supplemental Table 6: Regression models of medication usage by group. Values represent percent differences between individuals taking versus not taking the listed medications.

Medication Variables	*Single Variable*		*Medication Adjusted Medication Models*		*Medication Fully Adjusted Model*	
	(N=263)		(N=262)		(N=233)	
hsCRP	% Diff	95%CI	% Diff	95%CI	% Diff	95%CI
			Adj R^2 = .10		Adj R^2 = .31	
Insulin Medication	104%	(-5%,340%)	--	--	-53%	(-80%,9%)
Oral Hyperglycemic Medication	125%	(42%,258%)	73%	(8%,177%)	--	--
Anti-Hypertensive Medication	101%	(46%,177%)	66%	(19%,131%)	--	--
Statin Medication	61%	(12%,130%)	--	--	35%	(-3%,89%)
Anti-Inflammatory	9%	(-25%,60%)	--	--	--	--
Antacids	127%	(38%,273%)	--	--	--	--
Hormones	16%	(27%,86%)	66%	(9%,193%)	--	--

Fully adjusted model includes BMI, born in U.S.A, income, age, smoking status, gender.

Medication Variables	*Single Variable*		*Medication Adjusted Medication Models*		*Medication Fully Adjusted Model*	
	(N=263)		(N=260)		(N=230)	
IL-6	% Diff	95%CI	% Diff	95%CI	% Diff	95%CI
			Adj R^2 = .09		Adj R^2 = .29	
Insulin Medication	109%	(29%,238%)	77%	(10%,185%)	--	--
Oral Hyperglycemic Medication	49%	(10%,101%)	--	--	--	--
Anti-Hypertensive Medication	48%	(21%,82%)	35%	(10%,67%)	--	--
Statin Medication	28%	(2%,61%)	--	--	--	--
Anti-Inflammatory	-1%	(-22%,27%)	--	--	--	--
Antacids	65%	(20%,127%)	42%	(3%,96%)	--	--
Hormones	1%	(-25%,36%)	--	--	--	--

Fully adjusted model includes BMI, born in U.S.A, income, age, smoking status, gender
