# Supplementary material for: Highway proximity associated with cardiovascular disease risk: the influence of individual-level confounders and exposure misclassification
Source: Environ Health. 2013 Oct 3;12:84. doi: 10.1186/1476-069X-12-84 (PMC3907023; doi:10.1186/1476-069X-12-84)
Supplement: Additional file 8: Table S7 — Regression models of combustion exposure in the 2 weeks preceding the blood draw by group. Values represent percent differences between individuals with and without the exposure. [file 1476-069X-12-84-S8.pdf]

Supplemental Table 7. Regression models of combustion exposure in the 2 weeks preceding the blood draw by group. Values represent percent differences between individuals with and without the exposure.

Recent Combustion Variables	*Single Variable Models*		*Recent Combustion Exposure Adjusted*		*Recent Combustion Exposure Fully Adjusted*	
	(N=267)		*(N=262)*		*(N=237)*	
hsCRP	%Diff	95%CI	% Diff	95%CI	% Diff	95%CI
			Adj R^2 = .08		Adj R^2 = .32	
Cleaned Fireplace	31%	(-59%,324%)	--	--	--	--
Outdoor Grill	-48%	(-76%,11%)	-46%	(-75%,14%)	--	--
Burned Wood Leaves or Trash	7%	(-77,387%)	--	--	--	--
Burned Candles Incense or Oil	5%	(-26%,48%)	--	--	--	--
Wood or Coal Burning Stove	30%	(-79%,729%)	--	--	--	--
Around Smoke from Fire	4%	(52%,126%)	--	--	--	--
Operated Gasoline Powered Equipment	39%	(-45%,254%)	--	--	--	--
Operated Kerosene Powered Equipment	54%	(-52%,401%)	--	--	--	--
Smoked Tobacco	45%	(15%,115%)	--	--	--	--
In Home with Someone Smoking	61%	(10%,136%)	47%	(0%,116%)	--	--
In Car with Someone Smoking	0%	(-1%,0%)	--	--	--	--
At Work with Someone Smoking	35%	(-20%,128%)	--	--	--	--
Did Major Housecleaning	0%	(-1%,0%)	--	--	--	--
Metal Working	3%	(-62%,177%)	--	--	--	--
Cooking with Oil	66%	(21%,128%)	62%	(18%,123%)	53%	(15%,102%)
In Car with Engine Running in Garage	41%	(69%,540%)	--	--	--	--
On Highway 10 Minutes or More	-26%	(-48%,5%)	-32%	(-51%,-4%)	--	--
On Busy Streets 20 Minutes or More	-2%	(-31%,39%)	--	--	--	--
Walked Along Highway	-3%	(-30%,34%)	--	--	--	--

Recent Combustion Variables	*Single Variable Models*		*Recent Combustion Exposure Adjusted*		*Recent Combustion Exposure Fully Adjusted*	
	(N=267)		*(N=262)*		*(N=253)*	
**IL-6**	% Diff	95%CI	% Diff	95%CI	% Diff	95%CI
			Adj R^2 = .07		Adj R^2 = .28	
Cleaned Fireplace	-30%	(-59%,324%)	--	--	--	--
Outdoor Grill	-13%	(-76%,11%)	--	--	--	--
Burned Wood Leaves or Trash	-44%	(-37%,387%)	--	--	--	--
Burned Candles Incense or Oil	-13%	(-26%,48%)	--	--	--	--
Wood or Coal Burning Stove	-28%	(-79%,729%)	--	--	--	--
Around Smoke from Fire	-34%	(52%,126%)	-32%	(-59%,13%)		--
Operated Gasoline Powered Equipment	13%	(-45%,254%)	--	--	--	--
Operated Kerosene Powered Equipment	-2%	(-52%,401%)	--	--	--	--
Smoked Tobacco	28%	(-15%,115%)	--	--	--	--
In Home with Someone Smoking	2%	(10%,136%)	--	--	--	--
In Car with Someone Smoking	0%	(-1%,0%)	32%	(3%,70%)	--	--
At Work with Someone Smoking	-1%	(-20%,128%)	--	--	--	--
Did Major Housecleaning	0%	(-1%,0%)	--	--	--	--
Metal Working	2%	(-62%,177%)	--	--	--	--
Cooking with Oil	13%	(21%,128%)	--	--	19%	(-1%,45%)
In Car with Engine Running in Garage	51%	(-69%,540%)	--	--	--	--
On Highway 10 Minutes or More	-27%	(-48%,5%)	-19%	(-37%,4%)	--	--
On Busy Streets 20 Minutes or More	-29%	(-43%,11%)	-20%	(-38%,2%)	-22%	(-37%,-3%)
Walked Along Highway	-18%	(-33%,0%)	--		--	--

Fully adjusted model includes BMI, age, gender, smoking status.
