# Supplementary material for: Highway proximity associated with cardiovascular disease risk: the influence of individual-level confounders and exposure misclassification
Source: Environ Health. 2013 Oct 3;12:84. doi: 10.1186/1476-069X-12-84 (PMC3907023; doi:10.1186/1476-069X-12-84)
Supplement: Additional file 9: Figure S2 — Unadjusted analysis of associations between distance and hsCRP and IL-6 levels by age, Born USA and Smoking. Figure S3 Unadjusted analysis of associations between distance and hsCRP and IL-6 levels by gender and diabetic. [file 1476-069X-12-84-S9.zip › 1369630084906517_Figure S3.docx]

**Supplemental Figure 3.** Unadjusted analysis of associations between distance and hsCRP and IL-6 levels by gender and diabetic.

VS.

VS.

**Distance from I-93**

VS.

VS.

**Distance from I-93**
